# S-propargyl-cysteine promotes the stability of atherosclerotic plaque via maintaining vascular muscle contractile phenotype

**DOI:** 10.3389/fcell.2023.1291170

**Published:** 2024-01-24

**Authors:** Li Ping, Li Zhi-Ming, Zhang Bi-Shan, Zhu Lei, Yu Bo, Zhu Yi-Chun, Wang Ming-Jie

**Affiliations:** ^1^ Shanghai Key Laboratory of Bioactive Small Molecules, Department of Physiology and Pathophysiology, School of Basic Medical Sciences, The Innovative Research Team of High-level Local Universities in Shanghai, Fudan University, Shanghai, China; ^2^ Department of Vascular Surgery, Huashan Hospital, Fudan University, Shanghai, China

**Keywords:** atherosclerosis, S-propargyl-cysteine, plaque stabilization, VSMCs, phenotype switching

## Abstract

**Introduction:** Plaque rupture in atherosclerosis contributes to various acute cardiovascular events. As a new sulfide-containing donor, S-propargyl-cysteine (SPRC) has been reported to play a beneficial role in cardioprotection, potentially through its anti-inflammatory, anti-oxidative and anti-atherogenic activities. Our previous study observed an increase in eNOS phosphorylation in endothelial cells. However, it remains unclear whether SPRC influences vascular smooth muscle cells (VSMCs) within the plaque and if this effect contributes to plaque stabilization.

**Methods:** An atherosclerotic unstable plaque mouse model was established by subjecting ApoE^−/−^ mice to tandem stenosis of the right carotid artery along with a Western diet. Daily SPRC administration was conducted for 13 weeks. Plaque morphology and stability were assessed using MRI scanning and histopathological staining. In our *in vitro* studies, we stimulated human artery vascular smooth muscle cells (HAVSMCs) with platelet-derived growth factor-BB (PDGF-BB), both with and without 100 μM SPRC treatment. Cell phenotype was assessed using both Western blot and Real-time PCR. Cell proliferation was assessed using the BrdU cell proliferation kit and immunofluorescence of Ki-67, while cell migration was measured using scratch wound healing and transwell assay. MiR-143-3p overexpression and knockdown experiments were used to investigate whether it mediates the effect of SPRC on VSMC phenotype.

**Results and Discussion:** SPRC treatment reduced plasma lipid levels, increased collagen content and decreased cell apoptosis in atherosclerotic plaques, indicating improved plaque stability. Both *in vivo* and *in vitro* studies elucidated the role of SPRC in preserving the contractile phenotype of VSMCs through up-regulation of miR-143-3p expression. Furthermore, SPRC suppressed the pro-proliferation and pro-migration effects of PDGF-BB on HAVSMCs. Overall, these findings suggest that the inhibitory effect of SPRC on phenotype switch from contractile to synthetic VSMCs may contribute to its beneficial role in enhancing plaque stability.

## Introduction

Atherosclerosis, a chronic inflammatory vascular disease, is a common underlying factor in severe vascular diseases such as myocardial infarction (Reynolds and Smilowitz, 2023), stroke (Bonati et al., 2022), and aortic dissection (Yang et al., 2020). Multiple factors, including endothelial dysfunction, vascular remodeling, oxidative stress and inflammation, contribute to the development of atherosclerosis (Cirillo et al., 2023; Kong et al., 2022). Phenotype switching of vascular smooth muscle cells (VSMCs) is one of the most crucial factors in the pathogenesis of atherosclerosis. Studies have reported that α-smooth muscle actin (α-SMA) and smooth muscle protein 22α(SM22-α) serve as markers for the contractile phenotype of VSMCs, whereas osteopontin (OPN) is a marker for the synthetic phenotype (Furmanik et al., 2020; Zhang et al., 2022). VSMCs, which make up the tunica media of blood vessels (Tian et al., 2021), adopt a contractile phenotype in the physiological state to fulfill their function in maintaining vascular tone and normal blood pressure (Lacolley et al., 2017; Ouyang et al., 2021). However, when exposed to inflammatory factors or platelet-derived factors released after endothelial damage, VSMCs undergo dedifferentiation into a synthetic and inflammatory phenotype, often referred to as foam cell-like VSMCs (Doran et al., 2008; Li J. et al., 2023). Consequently, these cells exhibit excessive proliferation and migration from the tunica media to the intima of arteries (Chen et al., 2021; Wu et al., 2014). As the condition worsens, VSMCs within the plaque undergo gradual senescence and apoptosis, resulting in the formation of apoptotic debris that contributes to the formation of a calcified matrix (Durham et al., 2018). Furthermore, senescent VSMCs secrete increased levels of matrix metalloproteinases (MMPs), which degrade collagen fibers (Johnson, 2014). Consequently, the formation of a plaque with a thin fibrous cap that is prone to rupture occurs. In conclusion, the phenotype switching of VSMCs plays a crucial role in the stability of an atherosclerotic plaque.

S-propargyl-cysteine (SPRC) is a novel derivative of garlic extract that has been shown to significantly elevate hydrogen sulfide (H_2_S) levels in mammalian plasma by enhancing the expression and activity of cystathionine-γ-lyase (CSE), a metabolic enzyme responsible for H_2_S production (Huang et al., 2013; Wen and Zhu, 2015). H_2_S is a gasotransmitter with potent anti-inflammatory, anti-oxidative, and anti-apoptotic properties (Wang, 2012). Studies have also confirmed that H_2_S supplementation can reduce plaque size and decelerate the progression of atherosclerosis (Zhang et al., 2020). Our previous research has shown that SPRC can effectively delay the progression of atherosclerosis by up-regulating eNOS phosphorylation in endothelial cells, thereby mitigating cell damage (Li Z. M. et al., 2023). However, further research is needed to investigate the role of SPRC in mitigating plaque instability, specifically its potential impact on the process of VSMCs’ phenotype switching.

Therefore, our study aims to explore the impact of SPRC on the phenotype switching of VSMCs in both an *in vivo* atherosclerotic unstable plaque mouse model and an *in vitro* PDGF-BB stimulating cell model.

## Materials and methods

### Mice and atherosclerotic models

Male ApoE^−/−^ mice (inbred in a C57BL/6 background), aged 6–8 weeks, were obtained from GemPharmatech (Jiangsu, China). They were housed in SPF animal facilities under a 12-h light/12-h dark cycle at a controlled room temperature of 22°C–24°C and a relative humidity of 60%. The mice were randomly divided into five groups, including the regular diet sham (Control, Saline), model (Saline), low-dose SPRC (SPRC 20 mg/kg/d), high-dose SPRC (SPRC 80 mg/kg/d) and atorvastatin (10 mg/kg/d first-line medication as a positive control). All treatments were administered via intragastric administration once daily for a total of 13 weeks: 6 weeks before surgery and 7 weeks after surgery. In the sham group, the carotid artery was separated without ligation. The mice in the other four groups were fed a Western Diet (D12079B, Research Diet) and underwent right carotid artery tandem double stenosis 6 weeks after the initiation of Western Diet feeding (Chen et al., 2013). All animal protocols complied with relevant ethical regulations and received approval from the Laboratory Animal Experimentation Ethical Committee of Fudan University (20190221-060). Details of the establishment of the unstable atherosclerotic plaque mouse model are provided (Supplementary Figure S1).

### Magnetic resonance imaging

The mice were initially anesthetized with 3% isoflurane and maintained with 1%–2% isoflurane inhalation during the acquisition of the MRI signals. Subsequently, the mice were placed on the track of the magnetic resonance scanning device, and anesthesia was maintained with isoflurane. The respiratory rate of the mice was monitored in real-time to be maintained at 30 to 50 beats/min. The cervical blood flow in the mice was scanned using a small animal 11.7T Bruker Biospec high-field magnetic resonance imaging system (BioSpin; Bruker, Ettlingen, Germany). Respiration and heart rate were monitored using a balloon sensor and ECG trigger leads connected to an ECG/respiratory unit (Rapid Biomedical, Rimpar, Germany). The scan parameters were as follows: TR = 15 ms, TE = 2.5 ms, flip Angle = 20°, FOV = 2.56 × 2.56 × 2.56 cm^3^, acquisition matrix = 256 × 256×256, NA = 2, and the total scan time was 18 min. Sagittal and transverse Tl imaging was performed using a black-blood multi-slice spiral echo sequence. The sequence parameters of T1-weighted imaging were as follows: TR = 800 ms, TE = 7.5 ms, FOV = 2.56 × 2.56 cm^2^, acquisition matrix = 256 × 256, reconstruction matrix = 512 × 512, slice thickness = 0.5 mm, NA = 2, and the total scan time was 8 min. The total acquisition time per animal was approximately 60 min.

### Anatomic assessment and biochemical analyses

The mice were anesthetized with 1% sodium pentobarbital in the seventh week after surgery. Blood samples were obtained by puncturing the retro-orbital plexus with a capillary glass tube, collected into anticoagulation tubes containing EDTA-2K, and then plasma was obtained by centrifugation at 3500r/min for 15 min at 4°C. A cold whole-body PBS perfusion was performed to flush out the blood from the vascular system, and then a switch was made to a 4% paraformaldehyde perfusion to fix tissues and arteries. The bilateral carotid arteries, aortic arch and its main branches were dissected under a stereomicroscope. Artery samples for histology examination were stored in 4% paraformaldehyde at 4°C, while others were stored in liquid nitrogen for biochemical analyses. The enface images were captured using a camera (Fuji XT-3) equipped with a macro lens (Fuji XF 80 mm F2.8 R LM OIS WR). The levels of total cholesterol (TC), total triglycerides (TG), low-density lipoprotein (LDL-C), and high-density lipoprotein (HDL-C) in the plasma were measured using commercial kits (Nanjing Jiancheng, China).

### Morphology study of atherosclerosis plaque

To analyze the morphology of the plaques, we utilized Haematoxylin and Eosin (H.E.) Staining and Masson’s Trichrome Staining. The right carotid artery was fixed with 4% paraformaldehyde and embedded in paraffin. Sections were made, dehydrated using 100%, 90%,80% and 70% alcohol, and then stained with haematoxylin and eosin. Collagen content, fibrous cap thickness, and cholesterol crystals were also observed using Masson’s Trichrome Staining, which shows blue-stained collagen fibers and red-stained muscle fibers. Finally, all histological images were captured using an optical microscope (Olympus, Japan).

### Immunohistochemistry

Immunohistochemical staining was used to stain paraffin sections with α-SMA, SM22α, OPN, Collagen Ⅰ, Collagen Ⅲ and Collagen Ⅳ. The sections were incubated at room temperature in 10% goat serum for 20 min. Then, the corresponding primary antibodies were added and left overnight at 4°C. After removing the primary antibodies and washing with TBST, HPR-conjugated secondary antibodies were added to the sections and incubated for 45 min at room temperature. The sections were washed again, and a freshly prepared DAB working solution was used to detect positive expression. The captured images were analyzed using ImageJ software. All the primary antibodies used were purchased from Proteintech (United States), and their concentrations were summarized as follows:α-SMA (1:400, 14395-1-AP), SM22α (1:400, 10493-1-AP), OPN (1:800, 22952-1-AP), Collagen Ⅰ (1:4800, 66761-1-Ig), Collagen Ⅲ (1:4000, 22734-1-AP), Collagen Ⅳ (1:1,000, 55131-1-AP).

### Quantitative real-time PCR

Total RNA was extracted from animal tissues and cells using Trizol Reagent (Invitrogen, United States). The extracted RNA (0.5–1 μg) was then reverse transcribed into cDNA using a Reverse Transcription Kit (Toyobo, Japan) following the manufacturer’s instructions. Finally, the SYBR Green Master Mix (Toyobo, Japan) was added to cDNA samples to amplify and detect gene expression through qPCR. Supplementary Table S1 displays all the primers’ sequences we used.

### Cell culture

Human artery vascular smooth muscle cells (HAVSMCs, CRL-1999, ATCC, United States) were cultured in a humidified incubator at 37°C and 5% CO_2_ using HAVSMC growth medium (1,101, Sciencell, China). The cells were subcultured in 6-well plate dishes and divided into three groups: the control group, the PDGF-BB group and the PDGF-BB + SPRC group. The last two groups were treated with 50 ng/mL PDGF-BB (HY-P7055, MCE, China) for 24 h. Cells were pre-incubated with 100 μM SPRC for 6 h before PDGF-BB treatment in the PDGF-BB + SPRC group.

### Cell transfection assay

The hsa-miR-143-3p agomir/antagomir oligonucleotides and agomir/antagomir negative control were synthesized by Biotend (Shanghai, China). The hsa-miR-143-3p agomir sequence is 5′- UGA​GAU​GAA​GCA​CUG​UAG​CUC -3′, and the hsa-miR-143-3p antagomir sequence is 5′-CGC​AUU​AUU​ACU​CAC​GGU​ACG​A-3′. HAVSMCs were transfected with 150 nmol/L hsa-miR143-3p antagomir to decrease the cellular miR143-3p level or 50 nmol/L hsa-miR143-3p agomir to increase the cellular miR143-3p level. According to the manufacturer′s protocol, these oligonucleotides were transfected into cells using Lipofectamine RNAiMAX Reagent (Invitrogen, Thermo Fisher, United States).

### Cell proliferation assay

To determine cell proliferation, we used two methods: BrdU incorporation and Ki-67 immunofluorescence. For BrdU incorporation, the BrdU Cell Proliferation Kit (Millipore United States) was used. HAVSMCs were seeded in a 96-well plate at a density of 1 × 10^4^ cells/well. Subsequently, they were allowed to adhere, starved with basal medium and subjected to different treatments. After a 6-h incubation with the BrdU reagent, the cells were fixed, and the rate of cell proliferation was determined using an Anti-BrdU monoclonal antibody and the corresponding secondary antibody according to the manufacturer’s instructions. After adding the stop solution, the absorbance was immediately measured using a microplate reader at 450 nm for the determination wavelength and 550 nm for the reference wavelength.

Ki-67 is a marker of proliferative cell nuclei. HAVSMCs were seeded in glass bottom cell culture dishes at a density of 1 × 10^4^ cells/well. After 12 h, a basal medium with 1% FBS replaced the growth medium. Different treatments were administered to the cells after another 12 h. At the endpoint of the experiment, cells were fixed, permeabilized and incubated with blocking reagents (Beyotime). The Anti-Ki67 antibody (ab15580 Abcam UK) was added at 1:200 dilution to the dishes and left overnight at 4°C. The Alexa Fluor 488-conjugated secondary antibody (1:200 8878S CST United States) was then added and incubated in the dark for 2 h at room temperature. DAPI was used to stain the cell nuclei. Finally, fluorescence signals were detected using a confocal microscope (Zeiss Germany).

### Cell migration assay

To determine the migration ability of HAVSMCs, we utilized the Scratch Wound Healing Assay and the Transwell Migration Assay. In the Scratch Wound Healing Assay, cells were grown in the growth medium until the cell sheets covered 80% of the cultural area. A rectangle wound area was created by scratching the cells with a 200-μL pipette tip and washing away the debris with PBS. The cells were then treated differently for 24 h, and the migrated wound area was photographed using an optical microscope and measured using ImageJ software.

For the transwell migration assay, the 12-well transparent insert chamber (8 μm pore size, Corning United States) was pre-coated with 150 μL/well of collagen Ⅰ (A1048301 Gbico United States) for 2 h. Excess collagen Ⅰ was then removed, and 500 μL basal medium containing the corresponding treatment (PDGF-BB, PDGF-BB + SPRC, SPRC) was added to each well outside the chamber. Next, the cell suspension with a density of 1 × 10^4^ cells/well was seeded into the insert chamber, allowed to adhere and migrate through 8 μm pores, and adhere to the other side of the membrane facing the medium in the well with different treatment for 12 h. After washing the inserted chambers with PBS, cells remaining on the membrane were gently removed with a cotton swab, and the cells migrated through the membrane were fixed for 15 min with 4% paraformaldehyde and stained with Crystal Violet solution (Beyotime CHINA). Microscope images were captured, and ImageJ software were used for cell counting.

### Western blot

The BCA assay (Beyotime, CHINA) was utilized to measure the protein concentrations in HAVSMCs lysates. After being separated on a 10% SDS-polyacrylamide gel, proteins were transferred to the PVDF membranes (Millipore). The membranes were then blocked with 5% skim milk for 60 min at room temperature. Primary antibodies, including α-SMA (1:1,500, 14395-1-AP, Proteintech), SM22α (1:1,000, 60213-1-Ig, Proteintech), OPN (1:1,000, BM4208, Boster) and GAPDH (1:10000, 60004-1-Ig, Proteintech), were then added to the membrane, incubated overnight at 4°C. After washing with TBST for three times, the membranes were incubated with a Rabbit or Mouse HRP-conjugated secondary antibody (1:1,000 Beyotime) according to the species origin of the primary antibody for 90 min at room temperature. Finally, the enhanced chemiluminescence (ECL) method was used for visualization. ImageJ software was used for grayscale analysis.

### Statistical analysis

Data were presented as mean ± SEM and analyzed using SPSS 20.0 software (SPSS, Inc., Chicago, United States). The normal distribution of data was assessed for each data set before any comparison. For normally distributed data, differences between multiple groups were examined using one-way analysis of variance (ANOVA) followed by Fisher’s Least Significant Difference (LSD) test. For the data sets failed to pass normal distribution test, the Kruskal–Wallis test was employed. A *p*-value less than 0.05 indicates statistical significance. Specific *p*-values were marked in figures for those between 0.001 and 0.05. The statistical graphs were obtained by using GraphPad Prism 8.

## Results

### SPRC alleviates lipid deposition and plaque development in atherosclerotic mice

**FIGURE 1:**
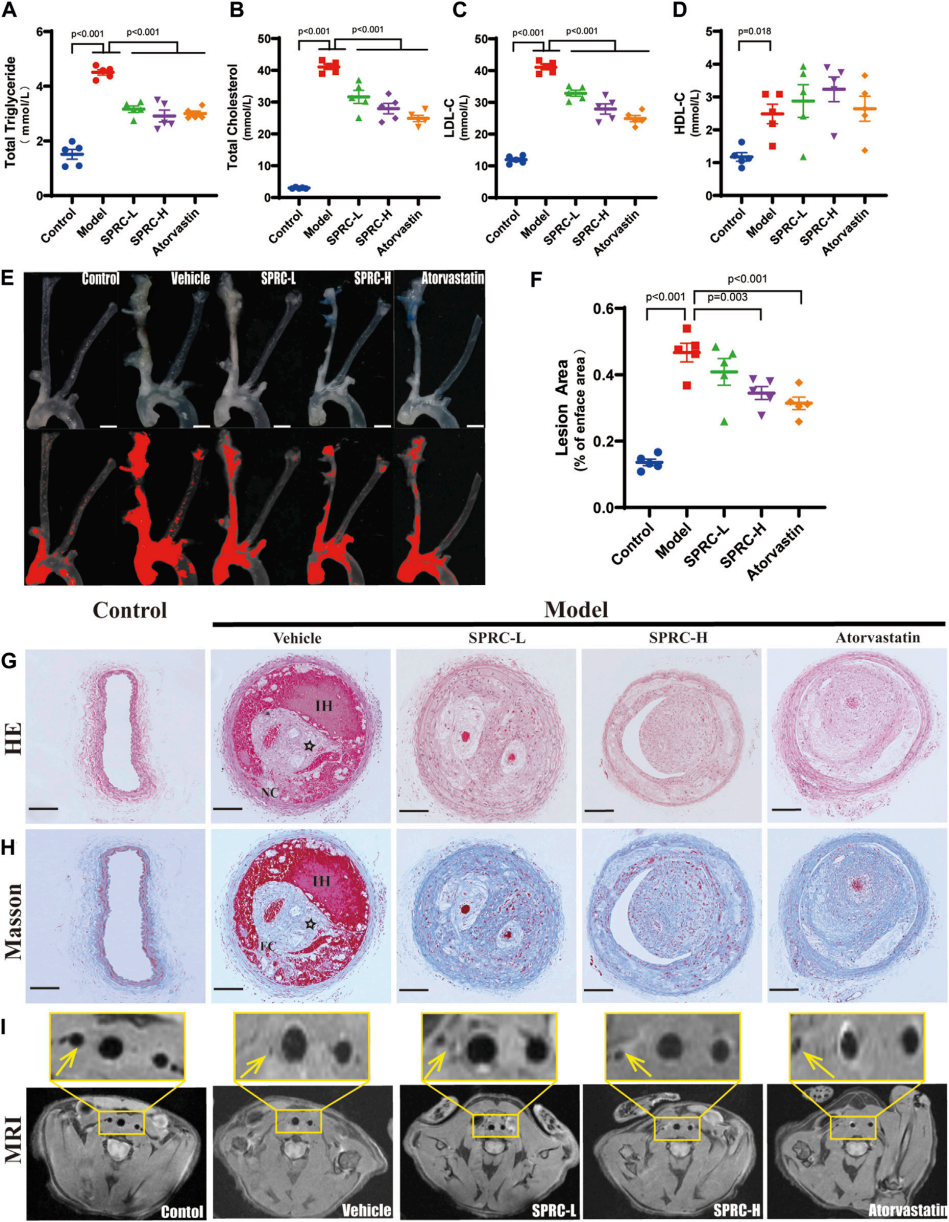
SPRC alleviates lipid deposition and plaque development in atherosclerotic mice. **(A–D)** Blood lipid level was elevated in model mice, while SPRC and Atorvastatin treatment alleviated the elevation. Mean ± SEM, n = 5. **(E)** Representative en-face photographs of the carotid artery and aorta arch dissected from mice of different groups. The red segment in the lower panel was marked by ImageJ software which represented the lesion area in blood vessels lost their transparency in the upper panel due to plaque formation. Scale bar, 3 mm. **(F)** SPRC and Atorvastatin treatment significantly decreased the percentage of lesion area compared to model mice. Mean ± SEM, n = 5. **(G, H)** Representative images of H.E. staining and Masson’s trichrome staining. For Masson’s trichrome staining, Collagen fibers were in blue and muscle fibers were in red. NC: necrotic core, FC: fibrous cap, IH: intraplaque hemorrhage, ☆: cholesterol crystal. Scale bar, 100 μm. **(I)** Representative images of T1-weighted MRI scanning. The right carotid artery which was indicated with a yellow arrow received tandem stenosis surgery while the left one served as self-control. The upper panel showed the partial enlargement of the rectangular area in the lower panel.

An unstable atherosclerotic plaque model was used to study the effect of SPRC on plaque stability. ApoE^−/−^ C57/BL6 male mice aged 6–8 weeks were subjected to right carotid artery tandem stenosis and fed a Western Diet that includes high cholesterol, according to modeling method described in the literature (Chen et al., 2013). Hyperlipidemia is the primary factor that leads to the development of atherosclerosis. Plasma levels of TG, TC, LDL-C and HDL-C were significantly elevated in the model group, whereas SPRC treatment alleviated the increase (Figures 1A–D). At the seventh week after surgery, bilateral carotid arteries and aortic arches were dissected to visualize the gross morphology of plaque, and the lesion area was evaluated. We found that the right carotid artery and aortic arch from the model mice were severely occluded by fatty lesions. However, a high dose of SPRC treatment significantly alleviated the occlusion (Figures 1E,F). The model mice exibited typical characteristics of unstable atherosclerotic plaques, such as necrotic core, thin fibrous cap and intraplaque hemorrhage, as demonstrated by H.E. staining and Masson’s trichrome staining (Figures 1G,H). Both SPRC and Atovastatin treatment mitigated plaque unstability. Additionally, magnetic resonance imaging (MRI) was used as a non-invasive method to monitor carotid artery stenosis. The black spot at the level of the carotid artery corresponded to the vascular lumen since the blood flow signal was magnetically saturated. The blood flow signal of the right carotid artery was barely visible in the model group, indicating severe artery stenosis. Administration of SPRC and Atorvastatin partially restored the vascular lumen (Figure 1I). Moreover, SPRC significantly restored the H_2_S level *in vivo* (Supplementary Figure S2).These results indicated that SPRC reduced blood lipid levels and attenuated the progression of unstable plaques in atherosclerotic mice.

### SPRC preserves contractile phenotype of VSMCs in atherosclerotic mice

**FIGURE 2:**
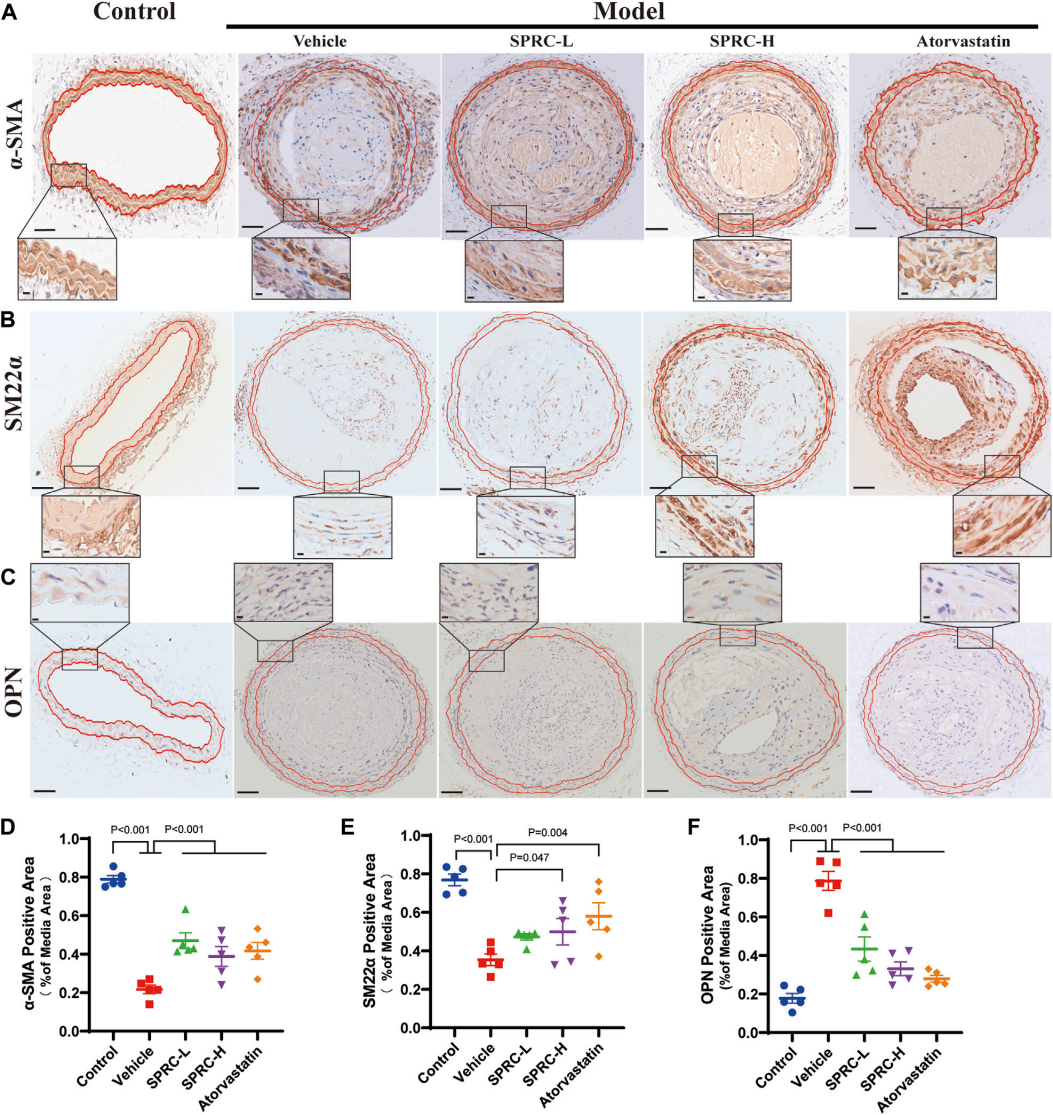
SPRC preserves contractile phenotype of VSMCs in atherosclerotic mice. **(A–C)** Representative images of α-SMA, SM22α and OPN immunohistochemical staining. The area enclosed by two red lines is the tunica media of the artery with the boundaries determined by H.E. staining of its adjacent continuous slice. Positive staining was shown in brown. The lower panel showed the partial enlargement of the rectangular area in the upper panel. Upper panel scale bar, 100 μm. Lower panel scale bar, 20 μm. **(D–F)** Quantitative analysis of α-SMA, SM22α and OPN positive area. Mean ± SEM, n = 5.α-SMA: smooth muscle actin alpha, SM22α: smooth muscle 22α, also known as Transgelin, OPN: osteopontin.

Remodeling of the phenotype of vascular smooth muscle cells (VSMCs) is one of the typical characteristics of atherosclerosis (Bennett et al., 2016). When exposed to fatty and inflammatory damage, VSMCs in the tunica media dedifferentiate into a synthetic type, leading to abnormal proliferation and formation of foam cells (Wang et al., 2019). Massive accumulation of foam cells leads to a larger lipid core, contributing to unstable plaque. To investigate the effect of SPRC on the phenotypic switching of VSMCs, immunohistochemistry staining of contractile or synthetic markers was performed. α-SMA and SM22α were used to observe the contractile phenotype, while OPN positive indicated the synthetic phenotype. The model group showed a significant reduction in α-SMA and SM22α positive areas (Figure 2A,B, D, E), while OPN positive areas were significantly increased (Figure 2C, F). Based on higher resolution images of IHC staining for OPN, the model group not only showed significantly increased OPN expression but also exhibited an increased cell number in the tunica media, as evidenced by Hematoxylin staining in blue. These results suggested that smooth muscle cells in the carotid artery of model mice had undergone phenotypic switching into synthetic cells and possessed ability to proliferate (Figure 2C). After 13 weeks of SPRC administration at a dose of 80 mg/kg/d, both SM22α and α-SMA positive areas were significantly increased, while OPN positive areas were reduced compared to the model mice (Figure 2A; 2D; 2B; 2E). Moreover, a lower dose of SPRC (20 mg/kg/d) was also effective in terms of α-SMA or OPN expression (Figure 2A, C, D, F). These results showed that SPRC could help maintain contractile phenotype of VSMCs, which may contribute to reducing areas of necrotic core and enhancing plaque stability.

### SPRC promotes plaque stability in atherosclerotic mice

**FIGURE 3:**
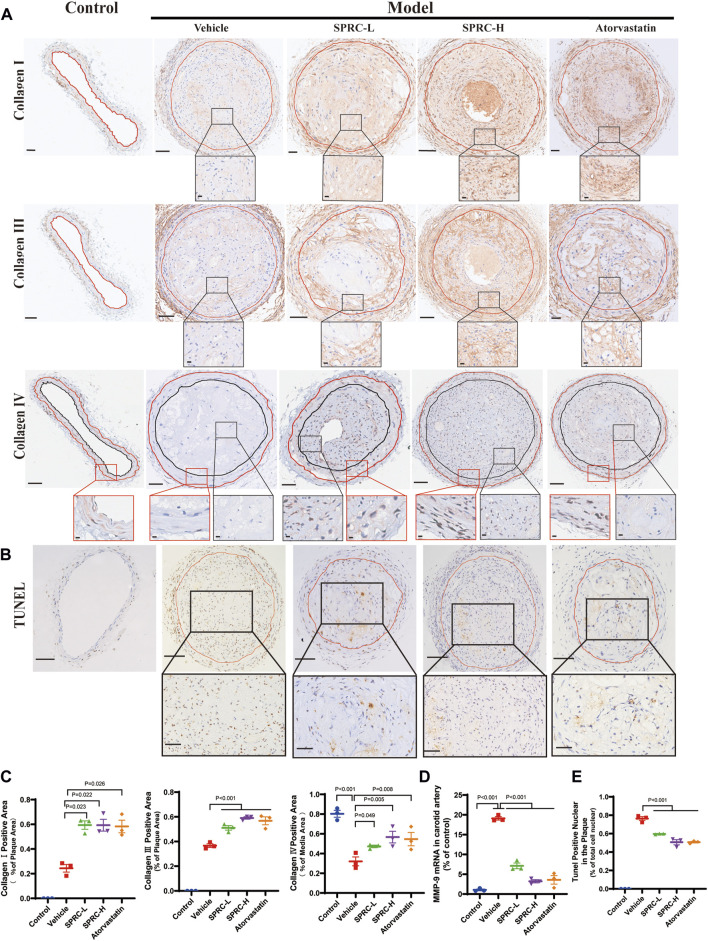
SPRC enhances plaque stability via increasing collagen content and reducing cell apoptosis *in vivo*. **(A)** Representative images of Collagen Ⅰ, Collagen Ⅲ and Collagen Ⅳ immunohistochemistry staining. The area enclosed by the red line indicates atherosclerotic plaque with the boundaries determined by H.E. staining of its adjacent continuous slice. Collagen Ⅰ/Ⅲ/Ⅳ positive was with brown color. The lower panel showed the partial enlargement of the rectangular area in the upper panel. Regarding Collagen Ⅳ immunohistochemistry staining, the red box showed a magnified view of the tunica media area of the carotid artery, and the black box showed a magnified view of the area within the plaque. Upper panel scale bar, 100 μm. Lower panel scale bar, 20 μm. **(B)** Representative images of cell apoptosis via TUNEL staining. The lower panel showed the partial enlargement of the rectangular area in the upper panel. The area enclosed by the red line indicates atherosclerotic plaque. Apoptotic cell nuclei were in brown, while healthy cell nuclei were in blue. Upper panel scale bar, 50 μm. Lower panel scale bar, 20 μm. **(C)** Quantitative analysis of Collagen Ⅰ and Collagen Ⅲ positive area. **(D)** MMP-9 mRNA expression in carotid artery was significantly elevated in model mice while SPRC treatment greatly reduced MMP-9 level. **(E)** The percentage of TUNEL positive apoptotic cells in the plaque was significantly decreased after SPRC treatment. Mean ± SEM, n = 3. MMP-9: matrix metalloproteinase-9.

The secretion of matrix metalloproteinases, such as matrix metalloproteinase-9 (MMP-9), could degrade collagen fibers, resulting in a thin fibrous cap (Sorokin et al., 2020). We observed that SPRC administration significantly increased collagen content in the plaque by two-fold compared to model mice (Figure 3A; 3C). Consistently, mRNA expression of MMP-9 was dramatically increased in the model group but significantly reduced after SPRC treatment (Figure 3D). IHC staining of Type Ⅳ Collagen, the main component of the basement membrane and a substance for MMP-9, was also performed to observe its change in atherosclerotic mice. As shown in Figure 3A (the lowest panel) the overall expression of collagen Ⅳ in the plaque was low. However, SPRC-treated mice exhibited more Type Ⅳ Collagen expression in the plaque compared to the untreated model group. For sake of more reliable statistical analyses, we measured the expression of collagen Ⅳ in the tunica media (the region falling within the manually added red and black coils according to H.E. staining). Results showed that the positive expression of collagen Ⅳ in untreated model group was significantly reduced to half of that in the control group, while SPRC treatment effectively increased collagen Ⅳ expression, which was consistent with the changes of the MMP-9 expression observed *in vivo*.

Apoptotic cells contribute to the formation of calcified matrix within plaques, which may lead to plaque rupture and thrombosis (Bennett et al., 2016). TUNEL staining showed massive accumulation of apoptotic cells within the plaque in model mice (Figure 3B). TUNEL-positive nuclei accounted for 80% of the total nuclei in model group (Figure 3E). In contrast, 80 mg/kg/d SPRC treatment significantly reduced the number of TUNEL-positive nuclei in the plaque compared to the model group. (Figure 3E). Collectively, our results demonstrated that SPRC enhanced plaque stability by increasing collagen content and reducing cell apoptosis.

### SPRC maintains contractile phenotype of HAVSMCs by up-regulating miR-143-3p

**FIGURE 4:**
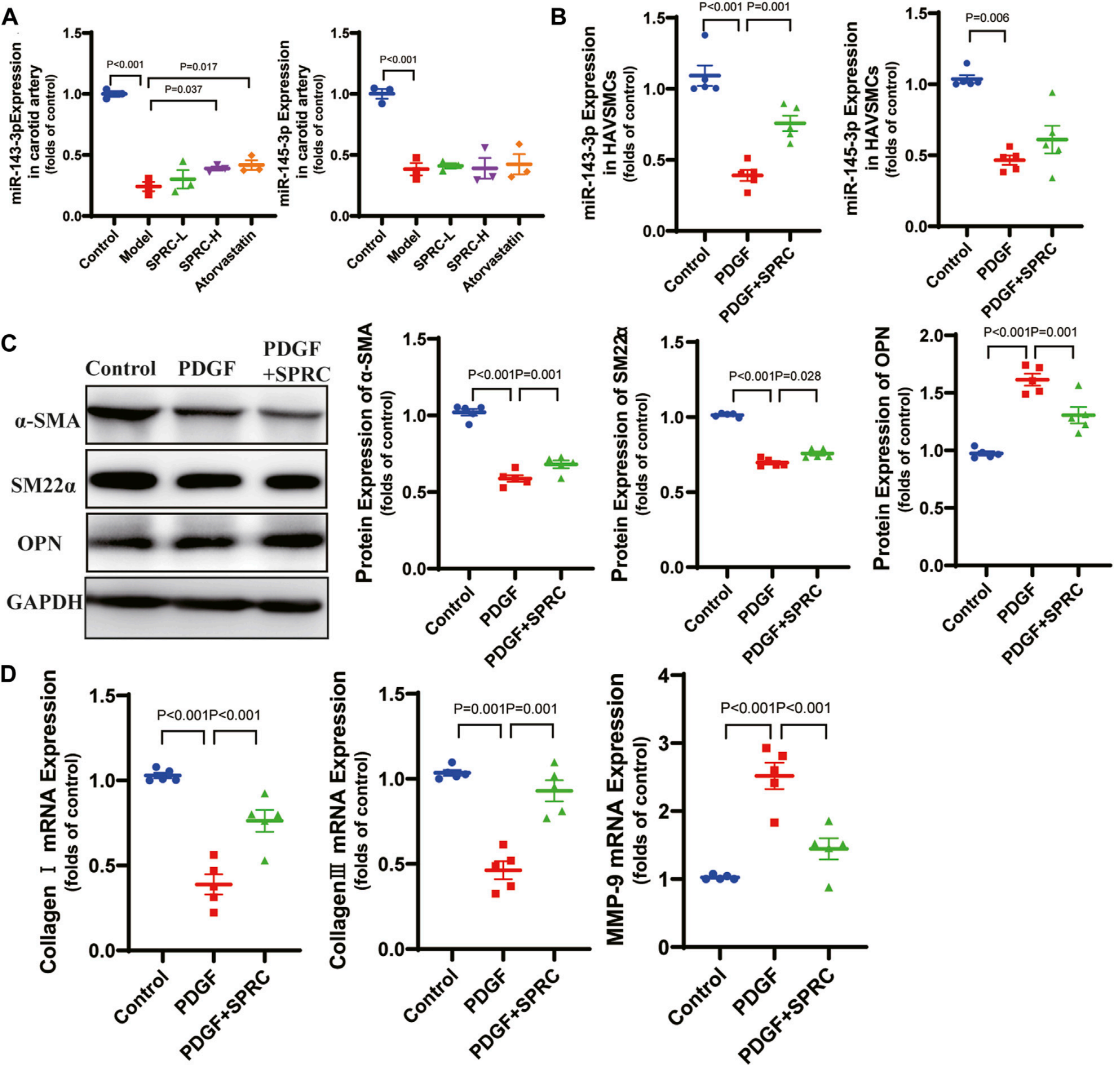
SPRC maintains contractile phenotype of HAVSMCs by up-regulating miR-143-3p. **(A)** Expression level of micro-RNA 143-3p/145-3p in the right carotid artery decreased in model mice, while SPRC treatment helped to restore miR-143-3p expression but not miR-145-3p expression. Mean ± SEM, n = 3. **(B)** Expression level of micro-RNA 143-3p/145-3p decreased in PDGF-BB-stimulated HAVSMCs, while SPRC treatment helped to restore miR-143-3p expression but not miR-145-3p expression. Mean ± SEM, n = 5. **(C)** Representative western blots of α-SMA, SM22α and OPN in HAVSMCs, and the corresponding quantitative analysis. Mean ± SEM, n = 5. **(D)** PDGF-BB-stimulation increased mRNA expression level of MMP-9 in HAVSMCs, resulting in a decrease in Collagen Ⅰ/Ⅲ mRNA expression. SPRC treatment mitigated these changes. Mean ± SEM, n = 5. MMP-9: matrix metalloproteinase-9.

According to previous reports, microRNA 143-3p/145-3p (miR-143-3p/145-3p) plays a crucial role in maintaining the contractile phenotype of VSMCs and preventing pathological vascular remodeling (Zhao et al., 2015). PDGF-BB is a widely used growth factor *in vitro* experiments to induce phenotype switching of VSMCs (Jung et al., 2021). We observed that 50 ng/mL PDGF-BB significantly increased cell viability in cultured HAVSMCs. So we used 50 ng/mL PDGF-BB for stimulation. We also confirmed that of SPRC ranging from 50 to 200 μM did not exhibit any potential toxicity (Supplementary Figure S3). We observed a significant decrease of expression of miR-143-3p/145-3p in the model group in both *in vitro* (Figure 4A) and *in vivo* (Figure 4B) experiments. SPRC administration upregulated miR-143-3p expression, but did not significantly affect miR-145-3p expression (Figures 4A,B). Protein expression of α-SMA and SM22α, two contractile phenotype markers, was significantly downregulated in PDGF-BB-stimulated HAVSMCs, but rebounded after 100 μM SPRC treatment. Protein expression of OPN, the synthetic phenotype marker, showed the opposite trend (Figure 4C). Moreover, mRNA expression of MMP-9 was significantly elevated in PDGF-BB-stimulated HAVSMCs, resulting in a decrease in Collagen Ⅰ/Ⅲ mRNA expression. SPRC treatment attenuated these changes (Figure 4D). These findings suggested that SPRC could maintain the contractile phenotype and function of vascular smooth muscle cells by up-regulating miR-143-3p.

### Overexpression and knockdown of intracellular miR-143-3p exert different effects on phenotype switching of HAVSMCs.

**FIGURE 5:**
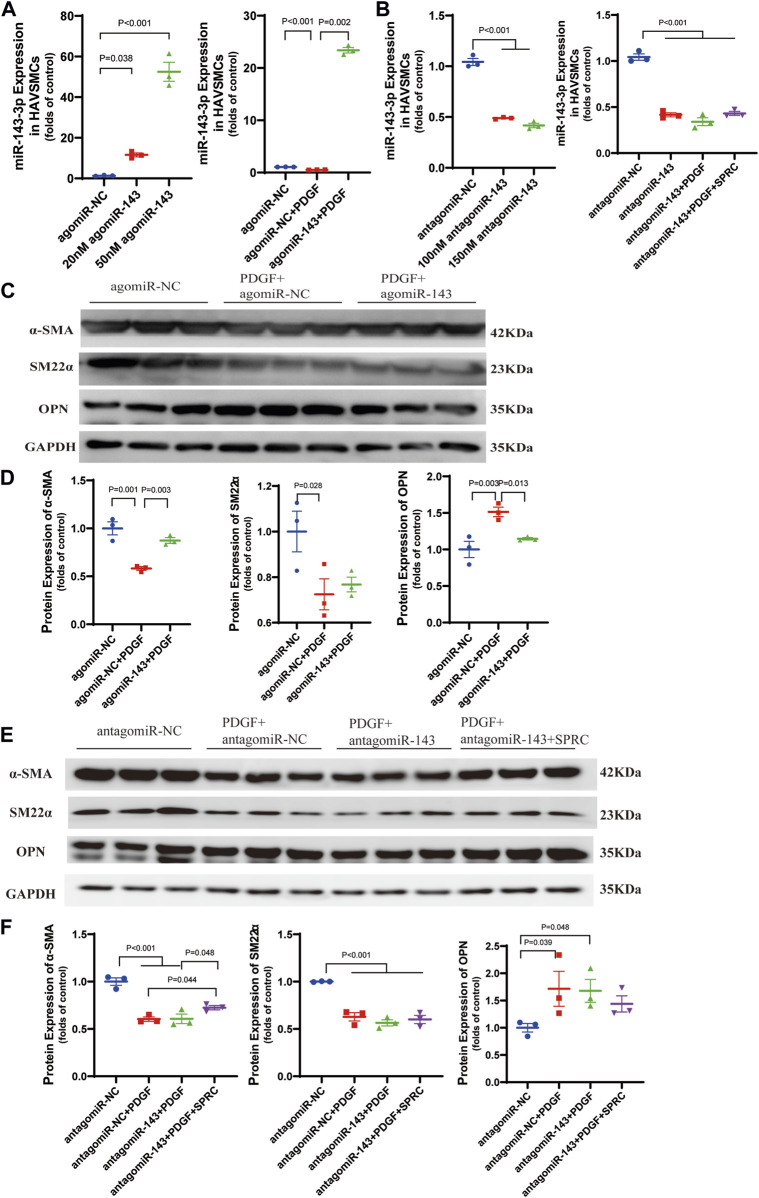
Overexpression and knockdown of intracellular miR-143-3p exert different effects on phenotype switching of HAVSMCs. **(A)** Expression level of micro-RNA 143-3p in HAVSMCs increased with agomiR transfection. **(B)** Expression level of micro-RNA 143-3p in HAVSMCs decreased with antagomiR transfection. **(C)** Representative western blots of α-SMA, SM22α and OPN in HAVSMCs in overexpression experiments. **(D)** Quantitative analysis of α-SMA, SM22α and OPN in HAVSMCs in overexpression experiments. **(E)** Representative western blots of α-SMA, SM22α and OPN in HAVSMCs in knockdown experiments. **(F)** Quantitative analysis of α-SMA, SM22α and OPN in HAVSMCs in knockdown experiments. Mean ± SEM, n = 3.

Based on the previous observation of the rebound effect of SPRC on miR-143-3p expression, we conducted miR-143-3p overexpression to investigate the role of elevated intracellular miR-143-3p level in maintaining the contractile phenotype. Both 20 and 50 nmol/L of agomiR-143-3p significantly increased the intracellular levels of miR-143-3p (Figure 5A). PDGF-BB reduced intracellular miR-143-3p expression to 50% in the agomiR NC group. However, with the introduction of 50 nmol/L of agomiR-143-3p, the expression level of miR-143-3p in the presence of PDGF-BB was still increased to 20-fold compared to the control group (Figure 5A). These results demonstrated that miR-143-3p overexpression could effectively counteract the reduction in miR-143-3p levels caused by PDGF-BB. Furthermore, we investigated whether overexpression of miR-143-3p could affect PDGF-BB-induced phenotypic switching. Following PDGF-BB stimulation, the expression levels of α-SMA and SM22α decreased to 50% compared to the control group, whereas the expression level of OPN increased 1.5 times (Figures 5C,D). Overexpression of miR-143-3p significantly increased α-SMA expression and decreased OPN expression, indicating that upregulation of miR-143-3p expression could mitigate the effect of PDGF-BB on phenotypic switching in HAVSMCs.

miR-143-3p knockdown experiments was conducted to verify whether the restoration of the contractile phenotype by SPRC depends on miR-143-3p expression. Both 100 and 150 nmol/L of antagomiR-143-3p significantly reduced intracellular miR-143-3p expression levels, with 150 nmol/L demonstrating a slightly stronger effect (Figure 5B). Therefore, we used150 nmol/L antagomiR-143-3p for subsequent experiments. Following treatment with 150 nmol/L of antagomiR-143-3p, the intracellular level of miR-143-3p was reduced to 50% compared to the control group, and further decreased when PDGF was introduced (Figure 5B). SPRC supplementation showed a tendency to increase the intracellular level of miR-143-3p, but the difference was not statistically significant, suggesting that SPRC was unable to effectively elevate the intracellular level of miR-143-3p when it had already been inhibited by antagomir (Figure 5B). Subsequently, we investigated whether SPRC could still inhibit PDGF-BB-induced phenotypic switching after miR-143-3p knockdown. The results indicated that regardless of whether miR-143-3p was knocked down or not (as observed in the antagomiR NC group), PDGF-BB treatment had a similar effect on reducing the expression levels of α-SMA and SM22α by 50%, while increasing the expression level of OPN to 1.7 times that of the control group. SPRC supplementation had distinct effects on the two contractile markers. The expression level of α-SMA was significantly increased, whereas SM22α remained unchanged. Although SPRC appeared to decrease the expression level of OPN, but the difference was not statistically significant (Figures 5E,F). These results suggested that the effect of SPRC on restoring the phenotype under PDGF-BB challenge is at least partially mediated by miR-143-3p.

SPRC alleviates abnormal proliferation and migration of HAVSMCs caused by PDGF-BB induced dedifferentiation.

**FIGURE 6:**
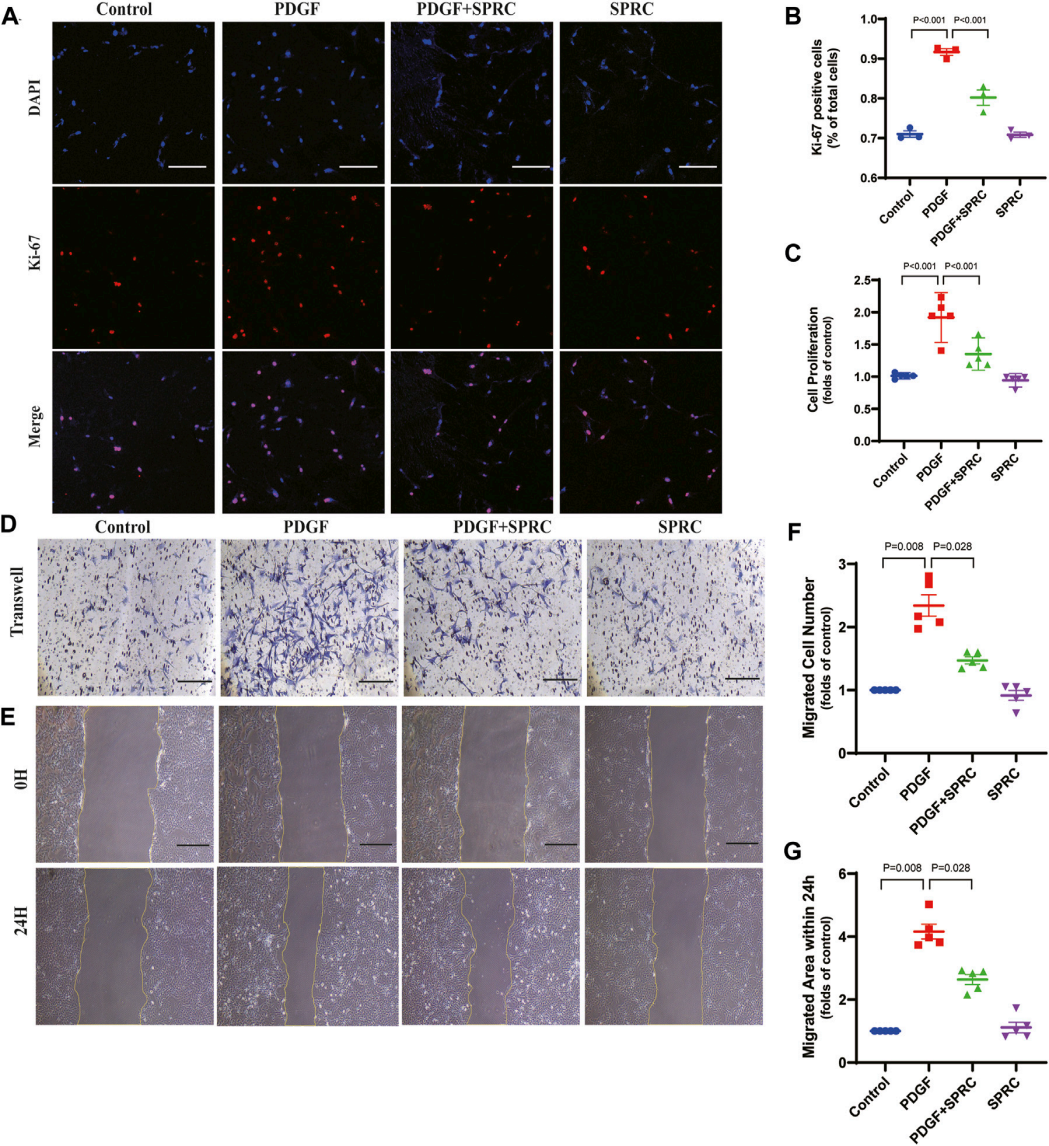
SPRC alleviates abnormal proliferation and migration of HAVSMCs caused by PDGF-BB induced dedifferentiation. **(A)** Representative immunofluorescence images of Ki-67 in HAVSMCs. Scale Bar, 200 μm. **(B)** Quantitative analysis of cell proliferation by using Ki-67 immunofluorescence assay. **(C)** Quantitative analysis of cell proliferation rate measured by BrdU incorporation assay. **(D)** Representative images of transwell migration assay. Scale Bar, 100 μm. **(E)** Representative images of scratch wound healing assay. Scale Bar, 100 μm. **(F)** Quantitative analysis of transwell migration assay. **(G)** Quantitative analysis of scratch wound healing assay. Mean ± SEM, n = 5.

Four *in vitro* experiments were conducted to investigate whether SPRC could prevent the excessive proliferation and migration of HAVSMCs induced by synthetic phenotype switching. Cell proliferation was observed using Ki-67 staining and BrdU cell proliferation assay, and cell migration was assessed through scratch wound healing assay and transwell migration assay. PDGF-BB-stimulation led to a significant increase in Ki-67 positive staining, a marker of proliferative cell nuclei, but this trend was suppressed by SPRC treatment (Figures 6A,B). BrdU assay revealed that PDGF-BB doubled cell proliferation rate, which was alleviated by SPRC treatment. However, SPRC did not directly affect cell proliferation under normal conditions in the absence of PDGF-BB (Figure 6C).

PDGF-BB could significantly elevate scratch wound healing speed, whereas SPRC treatment was effectively reduced the migrated area within 24 h compared to PDGF-BB-stimulated cells. However, SPRC did not affect the migration of HAVSMCs in the absence of PDGF-BB (Figure 6D, F). Transwell migration assay, which explores the migration behavior of cells without the influence of cell-cell adhesion, also showed the beneficial effect of SPRC in alleviating the increased migration speed of PDGF-BB-stimulated cells (Figure 6E, G). These findings illustrated that SPRC inhibited the PDGF-BB-induced abnormal proliferation rate and migration speed of HAVSMCs induced by PDGF-BB.

## Discussion

Garlic, an ancient healthy food, has been proved by modern medical research to possess anti-inflammatory, antioxidant, cardioprotective and hepatoprotective effects (Butt et al., 2009), primarily due to its main active ingredient, H_2_S. SPRC, a sulfide-containing compound and a structural analog of garlic extract, is reported to have beneficial effects in cardiovascular diseases due to its antioxidant, anti-inflammatory and anti-apoptotic properties (Wen and Zhu, 2015). For instance, SPRC was reported to attenuate cell apoptosis in a heart failure rat model by inhibiting caspase activation and increasing the ratio of Bcl-2/Bax (Huang et al., 2013). Moreover, SPRC has positive effects on cell viability and angiogenesis. Nevertheless, its role in anti-atherosclerosis and the molecular mechanism of plaque stabilization have yet to be investigated.

Atherosclerosis, a chronic inflammatory arterial disease, is closely associated with various risk factors, including an unhealthy diet rich in high cholesterol, alcohol abuse, and smoking (Li et al., 2018), among others. Formation of plaques is a typical pathological feature in atherosclerosis, resulting from the accumulation of fat, abnormal hemodynamics, oxidative stress injury (Falk, 2006), and other factors. Plaque rupture serves as a triggering factor for several critical cardiovascular events, including myocardial infarction and stroke (Bonati et al., 2022; Reynolds and Smilowitz, 2023). ApoE^−/−^ mice fed a Western diet for 4 months are commonly used as a mouse model of atherosclerosis (Johnson, 2014), however, the plaques developed in this model are typically stable. Considering this, we selected tandem stenosis of the right carotid artery in combination with a 13-week Western diet to establish an unstable atherosclerotic plaque model, which was first reported in 2013 (Chen et al., 2013). In our study, H.E. staining and Masson’s trichrome staining revealed the presence of a necrotic core, a thin fibrous cap and intraplaque hemorrhage within the plaque, indicating the successful establishment of an unstable plaque mouse model.

There is substantial evidence suggesting that a strong correlation between the phenotype change of VSMCs in response to vascular injury and plaque rupture (Sakakura et al., 2013). During the atherogenic process, various stimuli, including ox-LDL, PDGF-BB and IL-β, contribute to the phenotypic switch of VSMCs from a contractile to a synthetic state (Aherrahrou et al., 2020). Synthetic VSMCs are able to secrete extracellular matrix, proliferate and migrate from the tunica media to the intima, resulting in a significant thickening of the artery wall at the site of plaque formation. Subsequently, VSMCs within the plaque acquire phagocytic properties, leading to the uptake of lipids and their transformation into foam cells (Yap et al., 2021). Massive accumulation of foam cell derived from VSMCs and macrophages, along with inflammation, angiogenesis and outward remodeling, contributes to the vulnerability of an unstable plaque with thin fibrous cap (Wang et al., 2019). Reduced expression of contractile markers α-SMA and SM22α, along with an increased expression of the synthetic marker OPN, are typical indicators of phenotype switching in VSMCs(Allahverdian et al., 2018). Our study demonstrated that the administration of SPRC could preserve the contractile phenotype of VSMCs *in vivo*, with a protective efficacy similar to that of atorvastatin. MRI examination and en-face gross observation both confirmed that an 80 mg/kg/d dose of SPRC effectively mitigated artery blockage resulting from plaque progression.

Under continued exposure to inflammatory cytokines and oxidative stress, VSMCs undergo gradual senescence and secrete more matrix metalloproteinases, leading to a larger necrotic core and a reduction in collagen fibers in the plaque (Bennett et al., 2016; Sorokin et al., 2020). In our study, the model group exhibited a significant decrease in collagen content, upregulated expression of MMP-9, and an increase in apoptotic cells within the plaque. Remarkably, treatment with 80 mg/kg/d SPRC for 13 weeks substantially improved collagen content in the plaque and significantly decreased the rate of cell apoptosis. Moreover, calcification accelerates plaque rupture. Recent studies have highlighted that damaged VSMCs release vesicles containing alkaline phosphatase activity, which facilitates the deposition of calcium in the plaque (Bennett et al., 2016). Additionally, certain damaged VSMCs are able to transform into osteoblasts and secrete various osteogenic proteins, including Bone Morphogenetic Protein-2 (BMP-2) and B-Alkaline Phosphatase (B-ALP) (Du et al., 2011). Preventing calcification can enhance plaque stability and reduce arterial stiffness levels. Therefore, it remains to be investigated whether SPRC can inhibit calcification through reducing VSMCs damage.

MicroRNA, a class of small noncoding RNAs, play a key role in various cellular events, including cell proliferation and differentiation, which are closely correlated with VSMCs functions (Feng et al., 2019). For example, the overexpression of miR-130a significantly promotes VSMCs proliferation by inhibiting the expression of Growth Arrest-specific Homeobox (GAX) (de Gonzalo-Calvo et al., 2017; Pan et al., 2021). miR-199a meditates VSMCs differentiation by regulating the expression of FOXC2 (Cao et al., 2021). Importantly, the expressions of miR-143 and miR-145 are key regulators in maintaining the contractile phenotype of VSMCs (Cordes et al., 2009). In the present study, we observed a significant decrease in the expressions of both miR-143 and miR-145 in the model group. Interestingly, SPRC treatment is capable of improving the expression of miR-143, but it does not affect the expression of miR-145 both *in vivo* and *in vitro*. The modulation of miR-145 expression in VSMCs has been reported to be mediated by endothelial cell signaling (Zhao et al., 2015). In the current experimental setup, endothelium in unstable plaque is dysfunctional, and subcultured VSMCs alone do not receive any endothelial signals. This could explain why SPRC is effective in improving miR-143 expression but not miR-145.

We further investigated whether the effect of SPRC on maintaining the VSMCs phenotype is associated with increased intracellular levels of miR-143-3p through overexpression and knockdown experiments. Overexpression assays demonstrated that an elevated intracellular level of miR-143-3p effectively restored the expression of α-SMA and OPN in the presence of PDGF-BB, confirming the role of miR-143-3p in maintaining the contractile phenotype. Inhibition of intracellular miR-143-3p using antagomiR resulted in the failure of SPRC supplementation to restore SM22α expression, despite an increase in the expression of another contractile marker, α-SMA, in the presence of PDGF-BB. Regarding the synthetic marker OPN, SPRC appeared to reduce its elevated expression induced by PDGF-BB; however, no statistically significant difference was observed. By comparing these knockdown results with those of intact miR-143-3p, where SPRC effectively restored the expression of α-SMA, SM22α and OPN, it can be inferred that the effect of SPRC on restoring the phenotype under PDGF-BB challenge is at least partially mediated by miR-143-3p.

We also investigated the effect of SPRC on phenotype switching of VSMCs, as well as their proliferation and migration behavior *in vitro*. Our results indicated the protective effects of 100 μmol/L SPRC in maintaining the contractile phenotype of VSMCs and inhibiting excessive proliferation and migration induced by 50 ng/mL PDGF-BB. Nevertheless, *in vitro* experiments cannot fully replicate the changes that occur *in vivo*, where VSMCs interact with multiple factors and cooperate with various types of cells including endothelial cells, macrophages and fibroblasts during atherogenic progression. For example, the communication between endothelial cells and VSMCs is crucial for angiogenesis, wound repair, and vascular remodeling. Investigating the anti-atherogenic effects of SPRC in co-culture systems of ECs and VSMCs would be worthwhile in the future.

The current study confirmed that SPRC improves plaque stability. It reduces the plasma lipid levels and plaque size, enhances the collagen content and exerts anti-apoptotic activity in the plaque, and alleviates intraplaque hemorrhage. Our previous research reported that SPRC could delay the progression of atherosclerosis and promote eNOS phosphorylation in the endothelial cells (Li J. et al., 2023). However, the role of SPRC in smooth muscle cells within the plaque and whether this effect contributes to plaque stabilization remained unknown. In the present study, we found that SPRC preserves the contractile phenotype of VSMCs via upregulating the expression of miR-143 and suppressing abnormal proliferation and migration behavior of dedifferentiated cells. Consequently, SPRC is beneficial in delaying the progression of atherosclerosis and enhancing plaque stability.

## Data Availability

The original contributions presented in the study are included in the article/Supplementary Material, further inquiries can be directed to the corresponding author.

## References

[B1] Aherrahrou R. Guo L. Nagraj V. P. Aguhob A. Hinkle J. Chen L. (2020). Genetic regulation of atherosclerosis-relevant phenotypes in human vascular smooth muscle cells. Circ. Res. 127 (12), 1552–1565. 10.1161/CIRCRESAHA.120.317415 33040646 PMC7718324

[B2] Allahverdian S. Chaabane C. Boukais K. Francis G. A. Bochaton-Piallat M. (2018). Smooth muscle cell fate and plasticity in atherosclerosis. Cardiovasc. Res. 114 (4), 540–550. 10.1093/cvr/cvy022 29385543 PMC5852505

[B3] Bennett M. R. Sinha S. Owens G. K. (2016). Vascular smooth muscle cells in atherosclerosis. Circ. Res. 118 (4), 692–702. 10.1161/CIRCRESAHA.115.306361 26892967 PMC4762053

[B4] Bonati L. H. Jansen O. de Borst G. J. Brown M. M. (2022). Management of atherosclerotic extracranial carotid artery stenosis. Lancet Neurology 21 (3), 273–283. 10.1016/S1474-4422(21)00359-8 35182512

[B5] Butt M. S. Sultan M. T. Butt M. S. Iqbal J. (2009). Garlic: nature’s protection against physiological threats. Crit. Rev. Food Sci. Nutr. 49 (6), 538–551. 10.1080/10408390802145344 19484634

[B6] Cao Y. Cao Z. Wang W. Jie X. Li L. (2021). Microrna-199a-5p regulates foxc2 to control human vascular smooth muscle cell phenotypic switch. Mol. Med. Rep. 24 (3), 627. 10.3892/mmr.2021.12266 34212977 PMC8281299

[B7] Chen G. Xu H. Wu Y. Han X. Xie L. Zhang G. (2021). Myricetin suppresses the proliferation and migration of vascular smooth muscle cells and inhibits neointimal hyperplasia via suppressing tgfbr1 signaling pathways. Phytomedicine 92, 153719. 10.1016/j.phymed.2021.153719 34500301

[B8] Chen Y. C. Bui A. V. Diesch J. Manasseh R. Hausding C. Rivera J. (2013). A novel mouse model of atherosclerotic plaque instability for drug testing and mechanistic/therapeutic discoveries using gene and microrna expression profiling. Circ. Res. 113 (3), 252–265. 10.1161/CIRCRESAHA.113.301562 23748430

[B9] Cirillo M. Argento F. R. Attanasio M. Becatti M. Ladisa I. Fiorillo C. (2023). Atherosclerosis and endometriosis: the role of diet and oxidative stress in a gender-specific disorder. Biomedicines 11 (2), 450. 10.3390/biomedicines11020450 36830986 PMC9953736

[B10] Cordes K. R. Sheehy N. T. White M. P. Berry E. C. Morton S. U. Muth A. N. (2009). Mir-145 and mir-143 regulate smooth muscle cell fate and plasticity. Nature 460 (7256), 705–710. 10.1038/nature08195 19578358 PMC2769203

[B11] de Gonzalo-Calvo D. Cenarro A. Garlaschelli K. Pellegatta F. Vilades D. Nasarre L. (2017). Translating the microrna signature of microvesicles derived from human coronary artery smooth muscle cells in patients with familial hypercholesterolemia and coronary artery disease. J. Mol. Cell. Cardiol. 106, 55–67. 10.1016/j.yjmcc.2017.03.005 28342976

[B12] Doran A. C. Meller N. McNamara C. A. (2008). Role of smooth muscle cells in the initiation and early progression of atherosclerosis. Arteriosclerosis, Thrombosis, Vasc. Biol. 28 (5), 812–819. 10.1161/ATVBAHA.107.159327 PMC2734458 18276911

[B13] Du Y. Wang Y. Wang L. Liu B. Tian Q. Liu C. (2011). Cartilage oligomeric matrix protein inhibits vascular smooth muscle calcification by interacting with bone morphogenetic protein-2. Circ. Res. 108 (8), 917–928. 10.1161/CIRCRESAHA.110.234328 21350216

[B14] Durham A. L. Speer M. Y. Scatena M. Giachelli C. M. Shanahan C. M. (2018). Role of smooth muscle cells in vascular calcification: implications in atherosclerosis and arterial stiffness. Cardiovasc. Res. 114 (4), 590–600. 10.1093/cvr/cvy010 29514202 PMC5852633

[B15] Falk E. (2006). Pathogenesis of atherosclerosis. J. Am. Coll. Cardiol. 47 (8), C7–C12. 10.1016/j.jacc.2005.09.068 16631513

[B16] Feng S. Gao L. Zhang D. Tian X. Kong L. Shi H. (2019). Mir-93 regulates vascular smooth muscle cell proliferation, and neointimal formation through targeting mfn2. Int. J. Biol. Sci. 15 (12), 2615–2626. 10.7150/ijbs.36995 31754334 PMC6854371

[B17] Furmanik M. Chatrou M. van Gorp R. Akbulut A. Willems B. Schmidt H. (2020). Reactive oxygen-forming Nox5 links vascular smooth muscle cell phenotypic switching and extracellular vesicle-mediated vascular calcification. Circ. Res. 127 (7), 911–927. 10.1161/CIRCRESAHA.119.316159 32564697

[B18] Huang C. Kan J. Liu X. Ma F. Tran B. H. Zou Y. (2013). Cardioprotective effects of a novel hydrogen sulfide agent-controlled release formulation of s-propargyl-cysteine on heart failure rats and molecular mechanisms. PLoS One 8 (7), e69205. 10.1371/journal.pone.0069205 23874913 PMC3706411

[B19] Johnson J. L. (2014). Emerging regulators of vascular smooth muscle cell function in the development and progression of atherosclerosis. Cardiovasc. Res. 103 (4), 452–460. 10.1093/cvr/cvu171 25053639

[B20] Jung Y. Lee H. S. Ha J. M. Jin S. Y. Kum H. J. Vafaeinik F. (2021). Modulation of vascular smooth muscle cell phenotype by high mobility group at-hook 1. J. lipid Atheroscler. 10 (1), 99–110. 10.12997/jla.2021.10.1.99 33537257 PMC7838509

[B21] Kong P. Cui Z. Y. Huang X. F. Zhang D. D. Guo R. J. Han M. (2022). Inflammation and atherosclerosis: signaling pathways and therapeutic intervention. Signal Transduct. Target. Ther. 7 (1), 131. 10.1038/s41392-022-00955-7 35459215 PMC9033871

[B22] Lacolley P. Regnault V. Segers P. Laurent S. (2017). Vascular smooth muscle cells and arterial stiffening: relevance in development, aging, and disease. Physiol. Rev. 97 (4), 1555–1617. 10.1152/physrev.00003.2017 28954852

[B23] Li J. Li X. Song S. Sun Z. Li Y. Yang L. (2023a). Mitochondria spatially and temporally modulate vsmc phenotypes via interacting with cytoskeleton in cardiovascular diseases. Redox Biol. 64, 102778. 10.1016/j.redox.2023.102778 37321061 PMC10277590

[B24] Li M. Qian M. Kyler K. Xu J. (2018). Endothelial–vascular smooth muscle cells interactions in atherosclerosis. Front. Cardiovasc. Med. 5, 151. 10.3389/fcvm.2018.00151 30406116 PMC6207093

[B25] Li Z. M. Li P. Zhu L. Zhang Y. W. Zhu Y. C. Wang H. (2023b). S-propargyl-cysteine delays the progression of atherosclerosis and increases eNOS phosphorylation in endothelial cells. Sheng Li Xue Bao 75 (3), 317–327. 10.13294/j.aps.2023.0033 37340641

[B26] Ouyang C. Li J. Zheng X. Mu J. Torres G. Wang Q. (2021). Deletion ofulk1 inhibits neointima formation by enhancing kat2a/gcn5-mediated acetylation of tuba/α-tubulin *in vivo* . Autophagy 17 (12), 4305–4322. 10.1080/15548627.2021.1911018 33985412 PMC8726707

[B27] Pan W. Gao Y. Wan W. Xiao W. You C. (2021). Lncrna sammson overexpression suppresses vascular smooth muscle cell proliferation via inhibiting mir-130a maturation to participate in intracranial aneurysm. Neuropsychiatr. Dis. Treat. 17, 1793–1799. 10.2147/NDT.S311499 34113109 PMC8187098

[B28] Reynolds H. R. Smilowitz N. R. (2023). Myocardial infarction with nonobstructive coronary arteries. Annu. Rev. Med. 74 (1), 171–188. 10.1146/annurev-med-042921-111727 36179347

[B29] Sakakura K. Nakano M. Otsuka F. Ladich E. Kolodgie F. D. Virmani R. (2013). Pathophysiology of atherosclerosis plaque progression. Heart, Lung Circulation 22 (6), 399–411. 10.1016/j.hlc.2013.03.001 23541627

[B30] Sorokin V. Vickneson K. Kofidis T. Woo C. C. Lin X. Y. Foo R. (2020). Role of vascular smooth muscle cell plasticity and interactions in vessel wall inflammation. Front. Immunol. 11, 599415. 10.3389/fimmu.2020.599415 33324416 PMC7726011

[B31] Tan B. Jin S. Sun J. Gu Z. Sun X. Zhu Y. (2017). New method for quantification of gasotransmitter hydrogen sulfide in biological matrices by lc-ms/ms. Sci. Rep. 7 (1), 46278. 10.1038/srep46278 28406238 PMC5390247

[B32] Tian X. Zhou D. Zhang Y. Song Y. Zhang Q. Bu D. (2021). Persulfidation of transcription factor foxo1 at cysteine 457: a novel mechanism by which h2s inhibits vascular smooth muscle cell proliferation. J. Adv. Res. 27, 155–164. 10.1016/j.jare.2020.06.023 33318874 PMC7728583

[B33] Wang D. Yang Y. Lei Y. Tzvetkov N. T. Liu X. Yeung A. (2019). Targeting foam cell formation in atherosclerosis: therapeutic potential of natural products. Pharmacol. Rev. 71 (4), 596–670. 10.1124/pr.118.017178 31554644

[B34] Wang R. (2012). Physiological implications of hydrogen sulfide: a whiff exploration that blossomed. Physiol. Rev. 92 (2), 791–896. 10.1152/physrev.00017.2011 22535897

[B35] Wen Y. D. Zhu Y. Z. (2015). The pharmacological effects of s-propargyl-cysteine, a novel endogenous h_2_s-producing compound. Handb. Exp. Pharmacol. 230, 325–336. 10.1007/978-3-319-18144-8_16 26162842

[B36] Wu G. Cai J. Han Y. Chen J. Huang Z. Chen C. (2014). Lincrna-p21 regulates neointima formation, vascular smooth muscle cell proliferation, apoptosis, and atherosclerosis by enhancing p53 activity. Circulation 130 (17), 1452–1465. 10.1161/CIRCULATIONAHA.114.011675 25156994 PMC4244705

[B37] Yang K. Ren J. Li X. Wang Z. Xue L. Cui S. (2020). Prevention of aortic dissection and aneurysm via an aldh2-mediated switch in vascular smooth muscle cell phenotype. Eur. Heart J. 41 (26), 2442–2453. 10.1093/eurheartj/ehaa352 32428930

[B38] Yap C. Mieremet A. de Vries C. J. M. Micha D. de Waard V. (2021). Six shades of vascular smooth muscle cells illuminated by klf4 (krüppel-like factor 4). Arteriosclerosis, Thrombosis, Vasc. Biol. 41 (11), 2693–2707. 10.1161/ATVBAHA.121.316600 PMC8545254 34470477

[B39] Zhang H. Bai Z. Zhu L. Liang Y. Fan X. Li J. (2020). Hydrogen sulfide donors: therapeutic potential in anti-atherosclerosis. Eur. J. Med. Chem. 205, 112665. 10.1016/j.ejmech.2020.112665 32795766

[B40] Zhang S. Bei Y. Huang Y. Huang Y. Hou L. Zheng X. L. (2022). Induction of ferroptosis promotes vascular smooth muscle cell phenotypic switching and aggravates neointimal hyperplasia in mice. Mol. Med. 28 (1), 121. 10.1186/s10020-022-00549-7 36192693 PMC9528136

[B41] Zhao N. Koenig S. N. Trask A. J. Lin C. Hans C. P. Garg V. (2015). Microrna mir145 regulates tgfbr2 expression and matrix synthesis in vascular smooth muscle cells. Circ. Res. 116 (1), 23–34. 10.1161/CIRCRESAHA.115.303970 25323858 PMC4299754

