# Supplementary material for: S-propargyl-cysteine promotes the stability of atherosclerotic plaque via maintaining vascular muscle contractile phenotype
Source: Front Cell Dev Biol. 2024 Jan 24;11:1291170. doi: 10.3389/fcell.2023.1291170 (PMC10847265; doi:10.3389/fcell.2023.1291170)
Supplement: Supplementary file 1 [file DataSheet1.PDF]

Supplementary Material

1 Supplementary Figures and Tables.

1.1 Supplementary Figures

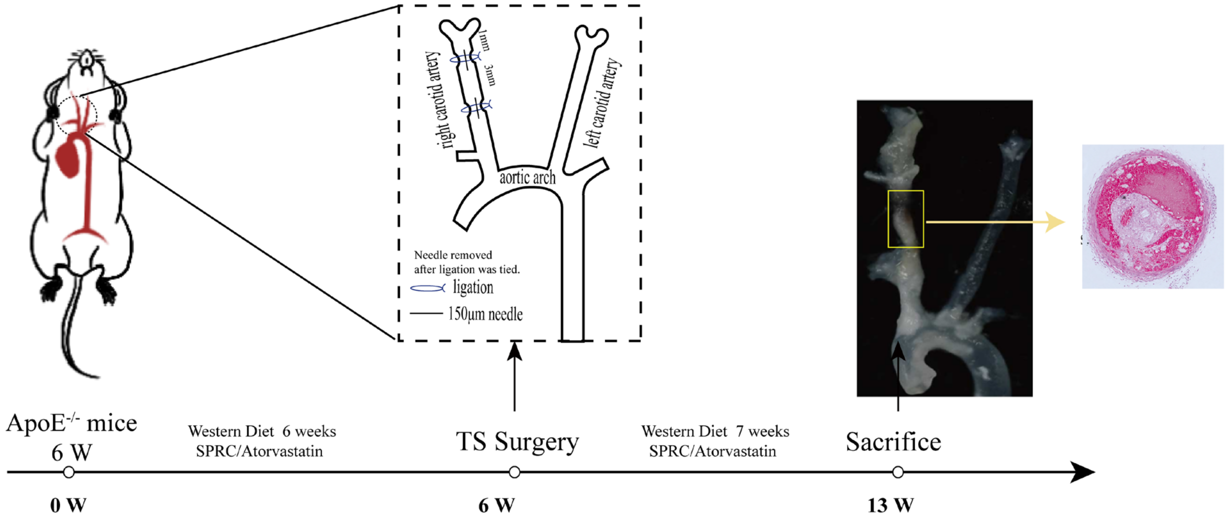

Supplementary Figure 1. The scheme of atherosclerotic mice model with unstable plaque and the intervention strategy. 6-week-old ApoE-/- mice were fed with a Western diet for 6 weeks, then received tandem stenosis surgery. In brief, both the right and left common carotid arteries were separated under a microscope after mice were anesthetized. Then, a 150-µm-diameter needle (Ethicon 8-0, silk blue, w1782) was placed alongside the right common carotid artery. At 1 mm away from the distal point of the carotid bifurcation and 3 mm away from the first ligation, we made two ligations with 6-0 polyester knitted fiber. Because the ligation tied the artery together with the needle, when the 150-µm-diameter needle was drawn out carefully, partial vascular stenosis was achieved. 7 weeks later, the animals were sacrificed, carotid arteries, aortic arch, thoracic aorta and its branches were isolated. The arterial segment between two ligations from the right carotid artery, marked by the yellow box in the scheme, was fixed, embedded and then H.E. staining was performed by using Paraffin slices to observe the pathological structure of the plaques.

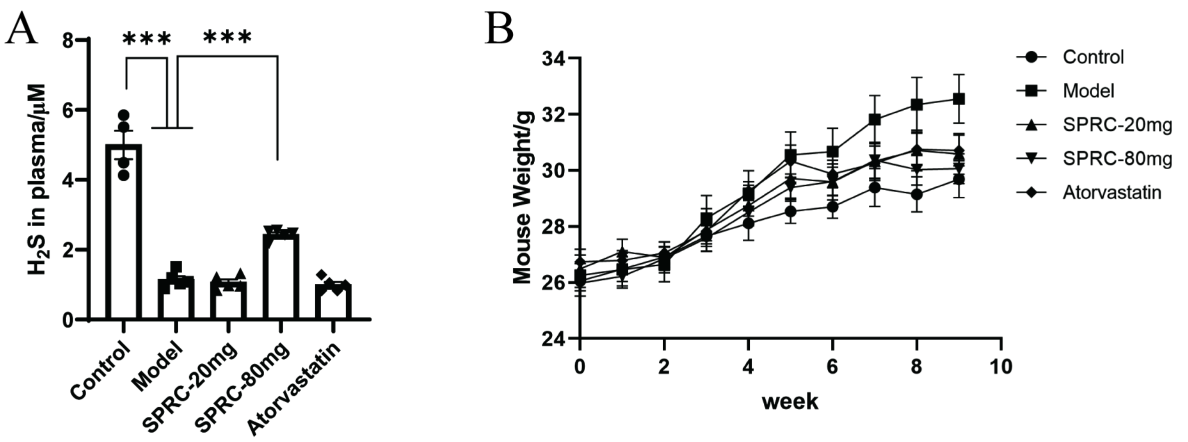

Supplementary Figure 2. The change of body weight and plasma H2S content in vivo. Liquid chromatography-tandem mass spectrometry method (LC-MS/MS) was used to measure H2S level in plasma according to literature(Tan et al.,2017). Briefly, monobromobimane (MBB), a fluorescent reagent that could react with thiol groups, was added in plasma to combine thiol groups in protein-bound hydrogen sulfide to produce derivative sulfide dibimane (SDB), then formic acid was used to stop reaction. After ultrafiltration, supernatant was collected for LC-MS/MS analysis. (A) H2S level in plasma. H2S content was significantly decreased in model group, while 80ng/kg/d SPRC treatment can restore plasma H2S level. (B) Body weight of mouse increased with time. SPRC administration had no significant effect on body weight.

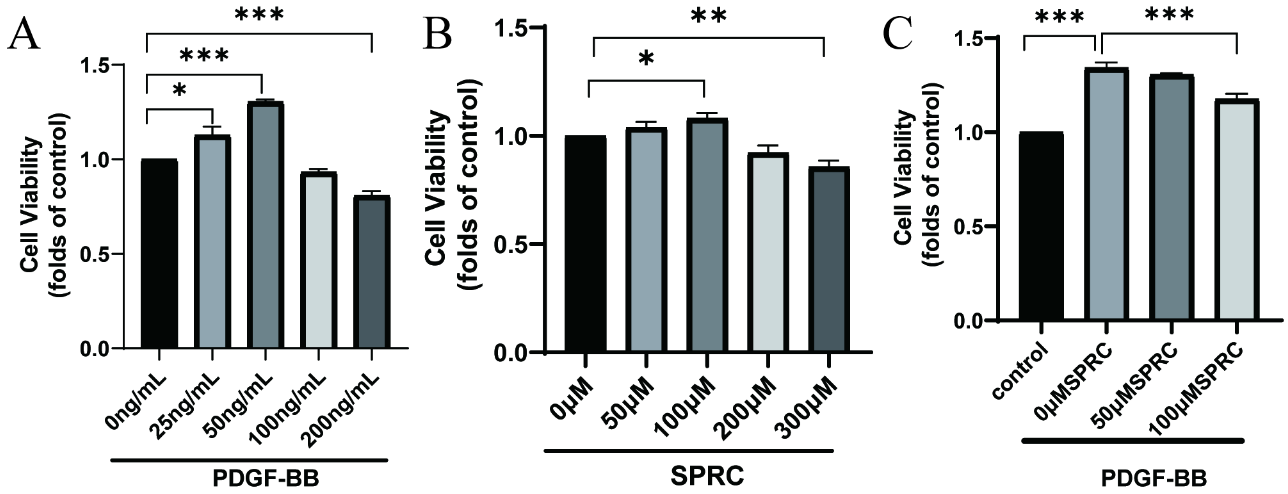

Supplementary Figure 3. Cell viability measurement. Cell viability was measured by CCK-8 kit (CK04, DOJINDO, Japan). Cell suspension was seeded in 96-well plates with a density of 1×10^4 cells/well, allowed to adhere and grow in complete medium for 12h. Then cells were synchronized with basal medium for another 12h, and then treated with different concentrations of PDGF-BB or SPRC at the following 24h. CCK-8 Reagent was then added and after 3-hour's incubation, absorbance was measured by a microplate reader at 450nm. (A) 50ng/mL PDGF-BB significantly increased the cell viability in cultured HAVSMCs. Hence, this concentration of PDGF-BB was used for stimulation. (B) There is no potential toxicity of SPRC ranging from 50 to 200 µM as cell viability in cultured HAVSMCs was not altered but 300µM SPRC significantly decreased cell viability which indicated that 300µM SPRC was harmful to HAVSMCs. (C) After the administration of 50 ng/mL PDGF-BB, treatment with 100 µM SPRC significantly suppressed excessive cell viability while exhibiting no inherent toxicity. Hence, 100 µM SPRC was administered for in vitro experiment.

1.2 Supplementary Tables

Gene	Sequence (5’-3’)
Universal R	GTGCAGGGTCCGAGGT
miR-143-3p F	TGAGACTGAGATGAAGCAC
miR-145-3p F	TGAGACATTCCTGGAAATAC
U6 F	CTCGCTTCGGCAGCACA
U6 R	AACGCTTCACGAATTTGCGT
Collagen I F	GAGGGCCAAGACGAAGACATC
Collagen I R	CAGATCACGTCATCGCACAAC
Collagen III F	GGAGCTGGCTACTTCTCGC
Collagen III R	GGGAACATCCTCCTTCAACAG
MMP-9 F	"TGTACCGCTATGGTTACACTCG "
MMP-9 R	GGCAGGGACAGTTGCTTCT
α-SMA F	GATCACCATCGGGAATGAACG
α-SMA R	CTTAGAAGCATTTGCGGTGGAC
GAPDH F	TGCCCCCATGTTCGTCA
GAPDH R	CTTGGCCAGGGGTGCTAA

Supplementary Table 1. List of sequences of the primers used for qPCR.
