# Supplementary figures and images for: S-propargyl-cysteine promotes the stability of atherosclerotic plaque via maintaining vascular muscle contractile phenotype
Source: Front Cell Dev Biol. 2024 Jan 24;11:1291170. doi: 10.3389/fcell.2023.1291170 (PMC10847265; doi:10.3389/fcell.2023.1291170)

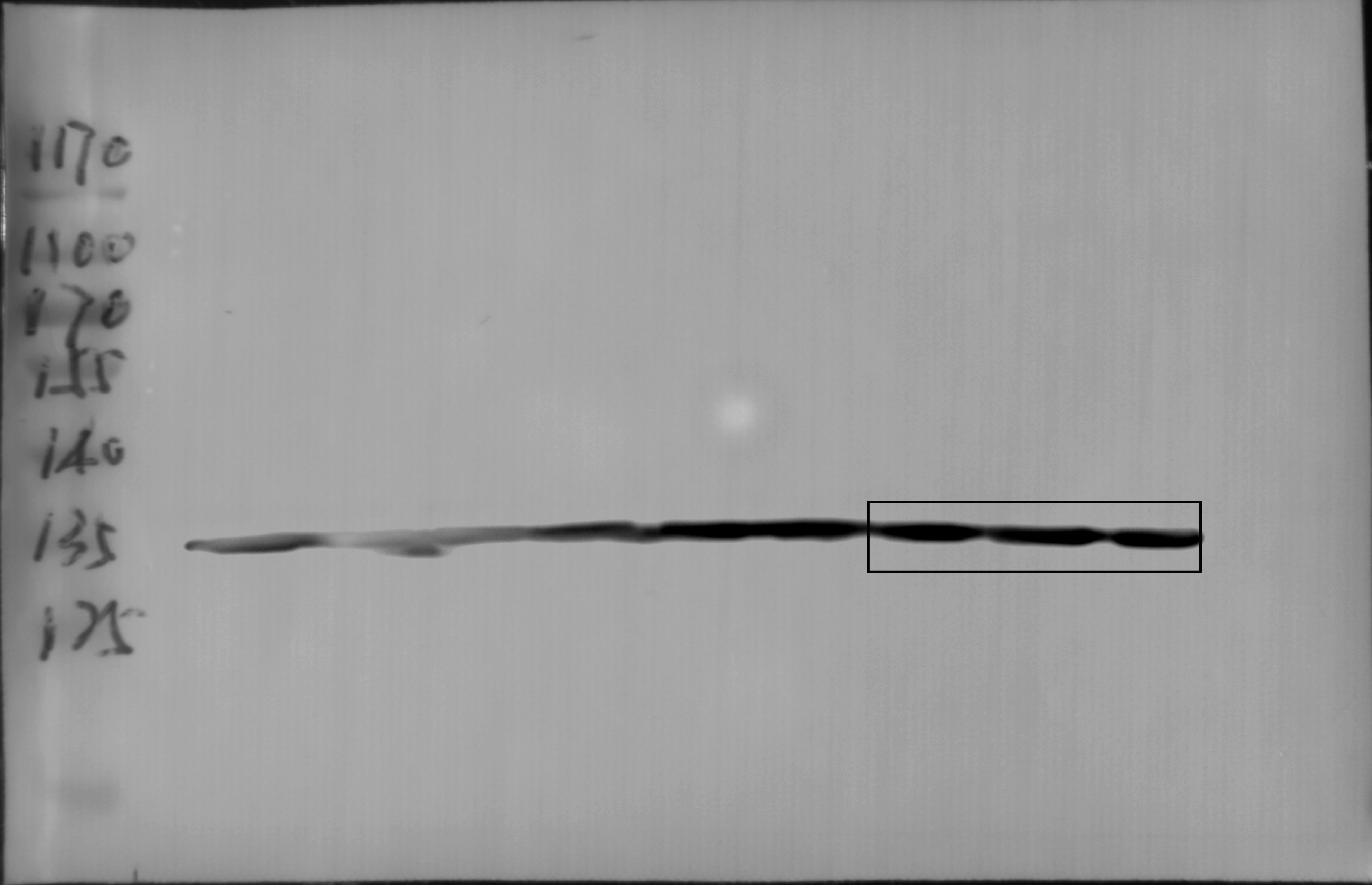

Supplement: Supplementary file 2 [file DataSheet2.ZIP › Raw data/figure 4 Western blot gels/GAPDH.png]

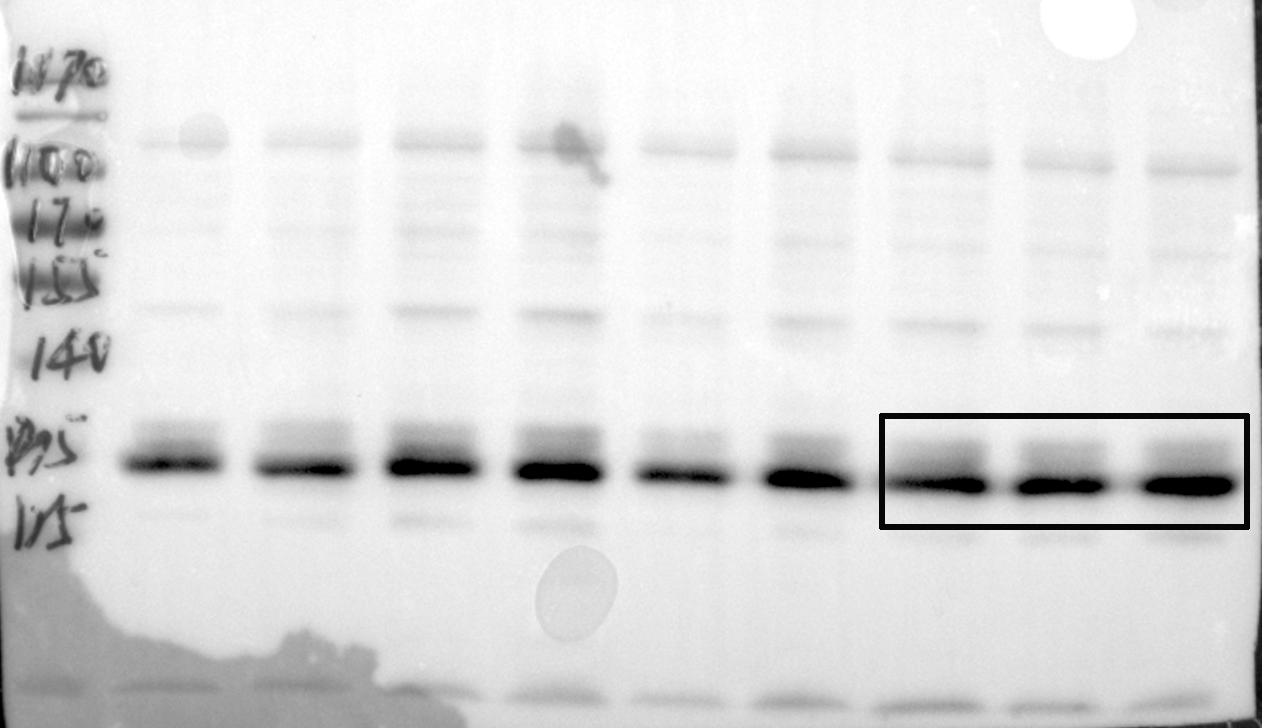

Supplement: Supplementary file 2 [file DataSheet2.ZIP › Raw data/figure 4 Western blot gels/OPN.png]

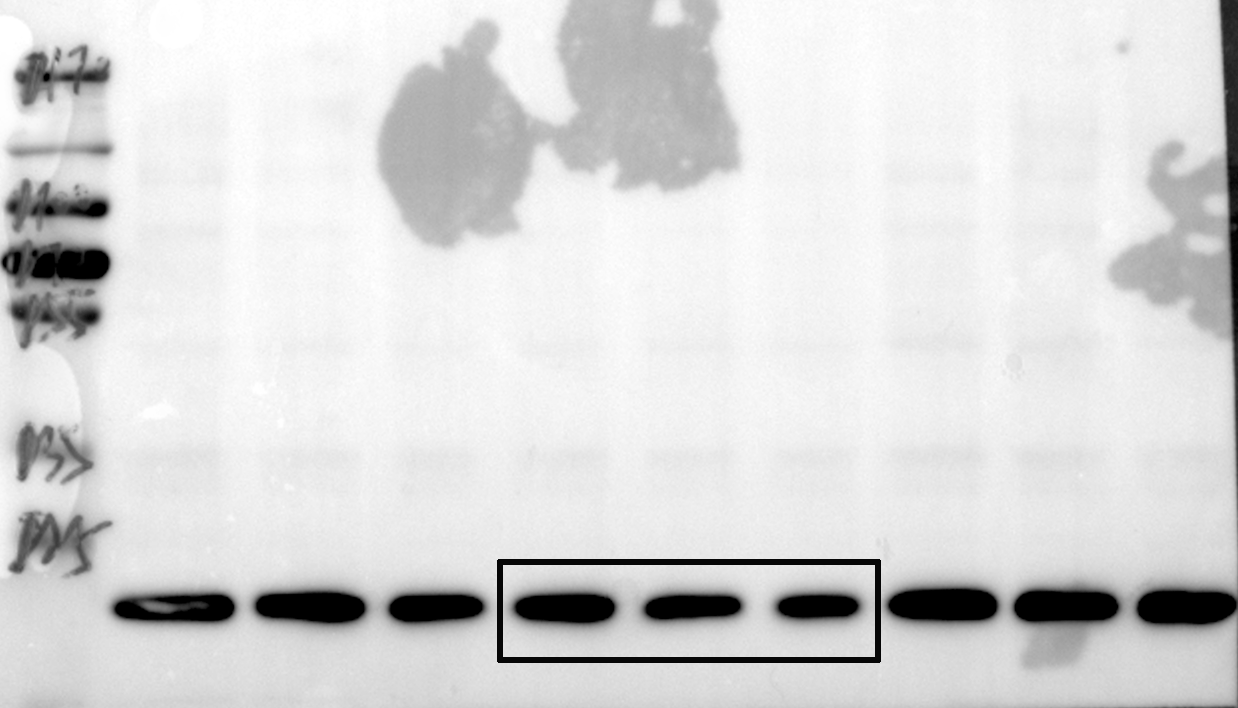

Supplement: Supplementary file 2 [file DataSheet2.ZIP › Raw data/figure 4 Western blot gels/SM22α.png]

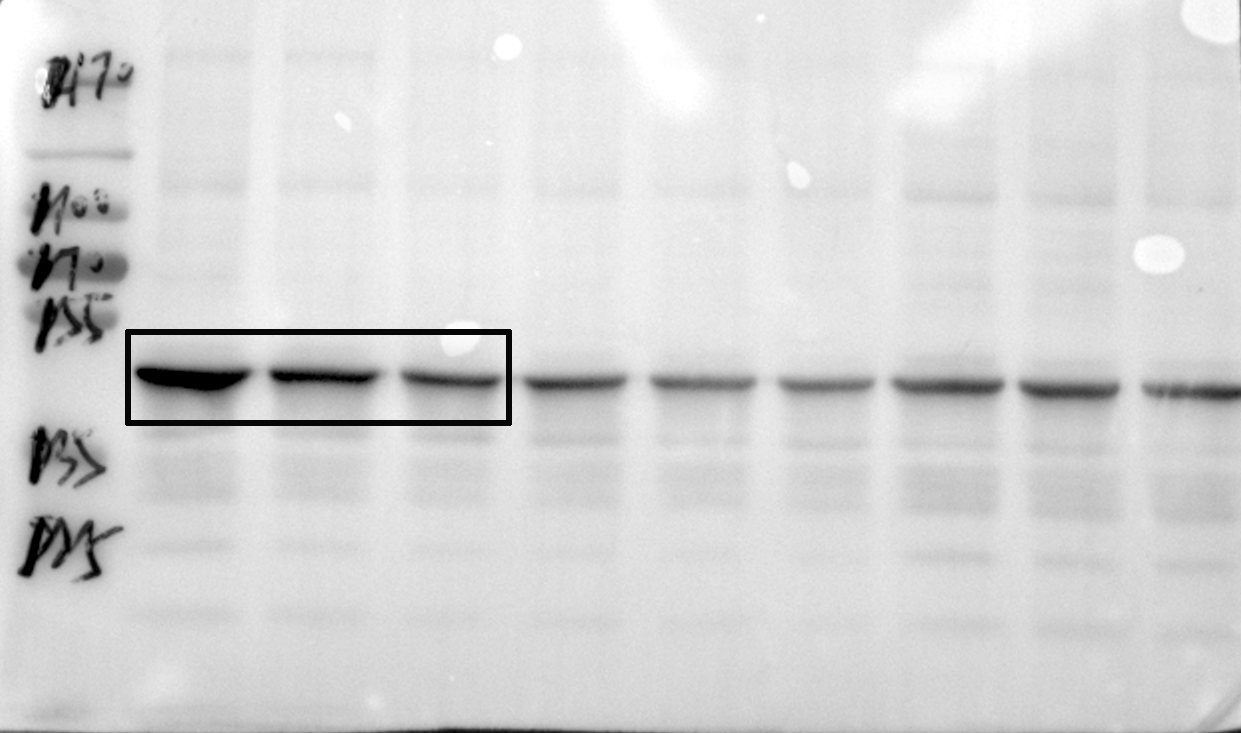

Supplement: Supplementary file 2 [file DataSheet2.ZIP › Raw data/figure 4 Western blot gels/α-SMA.png]
